# Shift towards larger diatoms in a natural phytoplankton assemblage under combined high-CO_2_ and warming conditions

**DOI:** 10.1093/plankt/fby018

**Published:** 2018-05-29

**Authors:** Scarlett Sett, Kai G Schulz, Lennart T Bach, Ulf Riebesell

**Affiliations:** 1 GEOMAR Helmholtz Centre for Ocean Research Kiel, Düsternbrooker Weg 20, Kiel, Germany; 2 Centre for Coastal Biogeochemistry, School of Environment, Science and Engineering, Southern Cross University, P.O. Box 157, Lismore, NSW, Australia

**Keywords:** ocean acidification, warming, diatoms, spring bloom, mesocosms

## Abstract

An indoor mesocosm experiment was carried out to investigate the combined effects of ocean acidification and warming on the species composition and biogeochemical element cycling during a winter/spring bloom with a natural phytoplankton assemblage from the Kiel fjord, Germany. The experimental setup consisted of a “Control” (ambient temperature of ~4.8 °C and ~535 ± 25 μatm *p*CO_2_), a “High-CO_2_” treatment (ambient temperature and initially 1020 ± 45 μatm *p*CO_2_) and a “Greenhouse” treatment (~8.5 °C and initially 990 ± 60 μatm *p*CO_2_). Nutrient replete conditions prevailed at the beginning of the experiment and light was provided at *in situ* levels upon reaching *p*CO_2_ target levels. A diatom-dominated bloom developed in all treatments with *Skeletonema costatum* as the dominant species but with an increased abundance and biomass contribution of larger diatom species in the Greenhouse treatment. Conditions in the Greenhouse treatment accelerated bloom development with faster utilization of inorganic nutrients and an earlier peak in phytoplankton biomass compared to the Control and High CO_2_ but no difference in maximum concentration of particulate organic matter (POM) between treatments. Loss of POM in the Greenhouse treatment, however, was twice as high as in the Control and High CO_2_ treatment at the end of the experiment, most likely due to an increased proportion of larger diatom species in that treatment. We hypothesize that the combination of warming and acidification can induce shifts in diatom species composition with potential feedbacks on biogeochemical element cycling.

## Introduction

Climate change, driven primarily by anthropogenic utilization of fossil fuels over the past 250 years, is changing sea surface temperatures (i.e. ocean warming) and seawater carbonate chemistry (i.e. ocean acidification). The IPCC 2014 projects an additional increase in sea surface temperature of 1–6 °C and a decrease in surface pH of ~0.3 units by the end of this century (Field *et al.*, 2014). Both of these environmental drivers are expected to affect marine primary producers (Riebesell and Tortell, 2011; Boyd and Brown, 2015) with potential consequences for marine biogeochemical element cycling in the future ocean (Riebesell *et al.*, 2009; Gehlen *et al.*, 2011; Rees, 2012).

Indirect effects of warming are expected through enhanced stratification of the water column and consequent alteration of nutrient supply and light availability, potentially affecting growth at the base of the marine food web and modulating energy transfer to higher trophic levels (Chust *et al.*, 2014). Below optimum temperatures for maximum metabolic rates (Boyd *et al.*, 2013), one of the direct effects of warming is the enhancement of metabolic activities (Eppley, 1972) which is considered to be more pronounced in heterotrophs than autotrophs (Pomeroy and Wiebe, 2001). Numerous laboratory and mesocosm-based experiments have been carried out to examine the effects of warming on natural plankton communities and some of the most common and recurrent patterns observed were increased primary production (PP) (Lewandowska *et al.*, 2012), shifts in species composition (Sommer *et al.*, 2007; Lassen *et al.*, 2010), shifts in the partitioning of organic matter into dissolved and particulate pools (Wohlers *et al.*, 2009) and decreased biomass build-up (Keller *et al.*, 1999; Sommer and Lengfellner, 2008a; O’Connor *et al.*, 2009; Lassen *et al.*, 2010; Wohlers-Zöllner *et al.*, 2012; Biermann *et al.*, 2014) with one exception (Taucher *et al.*, 2012). Taucher *et al.* (2012) suggested that the enhanced biomass build-up with warming during their experiments, contradictory to previous results, was most likely due to differences in the phytoplankton community composition.

In addition to warming, ocean acidification is also expected to affect marine ecosystems in the upcoming decades (Riebesell and Tortell, 2011). While studies looking at the effects of ocean acidification on natural phytoplankton communities have shown that there can be a fertilizing effect of increasing CO_2_ on PP (Hein and Sand-Jensen, 1997; Schippers *et al.*, 2004; Tortell *et al.*, 2008; Egge *et al.*, 2009; Engel *et al.*, 2013) others pointed towards a negative effect of rising proton (H^+^) concentration particularly on calcifying phytoplankton (Kottmeier *et al.*, 2014; Bach, 2015). At the community level, shifts in species composition were observed in some studies (Tortell *et al.*, 2002, 2008) with higher CO_2_ concentrations benefiting diatoms (Tortell *et al.*, 2008; Feng *et al.*, 2009; Yoshimura *et al.*, 2013). Most importantly, shifts in phytoplankton composition could affect the partitioning of organic carbon into the dissolved and particulate pools (Engel *et al.*, 2005; Riebesell *et al.*, 2007; Bellerby *et al.*, 2008; Kim *et al.*, 2011) which could have consequences for the export of organic carbon and CO_2_ sequestration through the biological carbon pump in the future. Ocean acidification and warming are two processes expected to occur simultaneously and thus their effects on natural phytoplankton communities should ideally be studied in combination (Hare *et al.*, 2007; Feng *et al.*, 2009; Kim *et al.*, 2011; Paul *et al.*, 2015; Riebesell and Gattuso, 2015).

By means of an indoor mesocosm experiment, we were able to follow the temporal development of a spring bloom containing a natural phytoplankton assemblage of a coastal eutrophic area in the Kiel Bight under control, ocean acidification (i.e. “High CO_2_”) and combined acidification and warming (i.e. “Greenhouse”) conditions. The increases in temperature and *p*CO_2_ applied in our experiment are within the range expected by the end of this century as projected by the IPCC 2014 (Field *et al.*, 2014).

## Materials and methods

### Experimental setup

The indoor mesocosm experiment was carried out between 12 January and 24 February 2012 at the GEOMAR Helmholtz Centre for Ocean Research Kiel. Six mesocosms (see end of section for details) with a volume of 1400 L each (1.5 m diameter, 1 m depth) were set up in temperature controlled rooms and filled simultaneously with seawater from Kiel Bight containing a representative late winter/early spring plankton community of bacteria, phytoplankton and microzooplankton. Although mesozooplankton was present in the water column at this time of year, most of it was probably trapped on the fine-sand filter collecting large detritus particles before filling the mesocosms. Dissolved inorganic nutrients at the beginning of the experiment were naturally available and therefore not manipulated. Each mesocosm had a computer-controlled light system equipped with full spectrum light bulbs (10 solar tropic T5 Ultra, 4000 K, 2 solar nature T5 Ultra, 9000 K, JBL) covering the full natural spectrum of photosynthetically active radiation (PAR: 400–700 nm). The light programme allowed for simulation of a daily triangular light curve with maximum light intensity (170–200 μmol photons m^−2^ s^−1^ at the surface) around noon (Brock, 1981) and a daily light dose increasing over time. Light was provided at conditions representing the mean of a mixed water column of ~10 m depth. Each mesocosm was equipped with a slowly rotating propeller which ensured gentle mixing throughout the experiment, allowing small particles to remain in suspension and bigger particles to sink through the water column (Sommer *et al.*, 2007). Mesocosms were set up in duplicates with the following treatments: “Control” (initial *in situ* temperature of 4.8 ± 0.3 °C and initial *in situ p*CO_2_ of 535 ± 25 μatm), “High CO_2_” treatment (initial temperature of 5.4 ± 0.3 °C and initially 1020 ± 45 μatm *p*CO_2_) and “Greenhouse” treatment (8.5 ± 0.4 °C and initially 990 ± 60 μatm *p*CO_2_). Elevated temperature and CO_2_ scenarios were chosen according to IPCC 2014 projections for the end of this century (Field *et al.*, 2014).

Temperature and salinity were measured every sampling day with a combined conductivity and temperature probe (WTW Germany). Samples for biogeochemical parameters and phytoplankton analysis were taken daily at 9 a.m. during the bloom period (Days 12–20) and every other day otherwise. Samples were taken from an intermediate depth (0.5 m below surface) either with a membrane pump directly into measuring vials or with a silicone tube by gravity into sample canisters. The canisters were gently mixed before any subsampling took place to ensure representative homogenous samples.

Low replication in the experimental setup (i.e. duplicates instead of triplicates per treatment) prevented us from conducting statistical analysis on our data. The low replication stemmed from unforeseen technical issues (i.e. propellers and light system failing) that, although immediately addressed during the experiment, could not be solved. The unbalanced design of the experiment (i.e. lack of a warming alone treatment) was due to logistical constraints of the available infrastructure. In view of this, we have applied a rather conservative approach in our data interpretation, remaining mostly descriptive and pointing out treatment effects only in cases where differences among treatments are rather obvious (i.e. no overlapping standard deviation). Nonetheless, we still believe our results provide valuable information about changes in community composition due to changing environmental conditions.

### Temperature and CO_2_ manipulations

Target temperature for the Greenhouse treatment (i.e. +4 °C above *in situ* conditions) was attained within 12 hrs after filling the mesocosms and remained constant throughout the experiment. For the CO_2_ manipulation, 25 L of seawater (same water used for filling the mesocosms) were aerated with pure CO_2_ (99.99%) for ~24 hrs before the manipulation. The CO_2_-saturated seawater was used to manipulate total dissolved inorganic carbon (DIC) in the High CO_2_ and Greenhouse treatments while leaving total alkalinity (TA) unaffected. The CO_2_-saturated seawater was added to the mesocosms at an intermediate depth with a silicone tube and the manipulation performed gradually over 2 days to avoid rapid changes and acute stress for the plankton community. During the CO_2_ manipulation, in all mesocosms, light conditions during the day were kept at 10% (17–20 μmol photons m^−2^ s^−1^) of the maximum surface irradiance to acclimate to the new carbonate chemistry conditions. Once target CO_2_ levels were attained (Experiment day 3), the regular daily light regime was started. Transparent floating lids (polyethylene, 0.3 mm thick) were placed on the water surface for the duration of the experiment to minimize gas exchange with the atmosphere. A gas tracer (N_2_O) was used to estimate gas exchange fluxes (Czerny *et al.*, 2013). Assuming that mesocosms in the same treatment behaved similarly in terms of gas exchange fluxes, N_2_O was only added to one mesocosm per treatment and any corrections (see below) were applied also to the replicate mesocosm.

### Carbonate chemistry

DIC samples were sterile filtered (0.2 μm) with a membrane pump at a flow rate of ~50 mL min^−1^ into 50 mL Schott Duran bottles with overflow and measured immediately using an infrared detection method for CO_2_ (AIRICA-automated infrared inorganic carbon analyzer) with ~2 μmol kg^−1^ precision. N_2_O was used as a gas tracer to estimate air/water gas exchange of CO_2_ and the estimated loss/gain in CO_2_ was used to calculate the biologically driven changes (i.e. photosynthesis and respiration) on DIC concentrations (i.e. DIC_bio_). DIC concentrations presented in this study are therefore representative for biological consumption, unless stated otherwise.

Samples for TA were filtered through GF/F filters and measured within 2 days in a Metrohm Basic Titrino 794 titration device according to Dickson *et al.* (2003). Measurements for TA and DIC were corrected with certified reference material (Dickson *et al.*, 2003). Calculation for carbonate chemistry speciation was carried out with the programme CO2SYS (Lewis and Wallace, 1998) from measured DIC, TA, temperature, salinity and nutrients (i.e. PO_4_ and Si(OH)_4_), using the dissociation constants by Mehrbach *et al.* (1973) as refitted by Dickson and Millero (1987).

### Biological and chemical analyses

Dissolved inorganic nutrients nitrate (NO_3_^−^), phosphate (PO_4_^3−^) and silicic acid (Si(OH)_4_) as well as dissolved organic carbon (DOC), nitrogen (DON) and phosphorus (DOP) were filtered through pre-combusted (450 °C, 6 hrs) GF/F filters with a membrane pump at a rate of ~100 mL min^−1^ directly into vials. Except for DOC samples, which were fixed with 85% phosphoric acid (0.5% *v*/*v* final concentration) and refrigerated, all samples were frozen at −20 °C until analysis. Nutrients were measured on a segmented flow autoanalyzer (SEAL QuAAtro) according to Hansen and Koroleff (1999). DOC samples were analysed using the high-temperature catalytic oxidation method on a Shimadzu TOC-V analyzer according to Wurl and Min Sin (2009). The method was calibrated against deep-sea reference material provided by the University of Miami. DON/DOP samples were pressure-cooked with Oxisolv (MERCK) and once oxidized, both organic nitrogen and phosphorus were measured as nitrate and phosphate according to Hansen and Koroleff (1999). DON/DOP concentrations were obtained by subtracting the respective inorganic nutrient concentrations.

Samples for particulate organic carbon (POC), nitrogen (PON) and phosphorus (POP) were filtered at low vacuum (200 mbar) onto pre-combusted (450 °C, 6 hrs) GF/F filters and stored at −20 °C until analysis. Filters for POC/PON were fumed with hydrochloric acid (37% HCl) for 2 hrs to remove all inorganic carbon and subsequently dried overnight at 60 °C. Samples were analysed with a EuroEA analyzer according to Sharp (1974). POP filters were pressure-cooked for 30 min with Oxisolv (Merck) and POP oxidized to orthophosphate, which was measured photometrically according to Hansen and Koroleff (1999). Samples for particulate biogenic silica (BSi) were filtered at low vacuum (200 mbar) onto cellulose acetate filters and stored at −20 °C until analysis. Samples were cooked in a NaOH solution for 2 hrs and 15 min at ~85 °C to extract all biogenic silica into solution and then analysed as silicate according to Hansen and Koroleff (1999).

Initial POM values were calculated by averaging concentrations of all treatments on the first 2 days of the experiment (±standard deviation). Build-up of POM was calculated by subtracting the average of the first 2 days (once target *p*CO_2_ levels were established) from measurements at each experimental day. In most cases, maximum POM build-up was observed after nutrient depletion and maximum levels lasted for more than 2 experimental days.

### Phytoplankton composition analysis

Phytoplankton species composition was analysed by pigment analysis [high-pressure liquid chromatography (HPLC)], size categorization by flow cytometry and Utermöhl microscopy. HPLC samples were filtered onto pre-combusted (450°C, 6 hrs) GF/F filters and stored at −80 °C until extraction. Photosynthetic pigments were separated via HPLC with 100% acetone (HPLC grade) according to the method of Van Heukelem and Thomas (2001). Phytoplankton composition was calculated with the programme CHEMTAX (Mackey *et al.*, 1996) which estimates taxon-specific contributions to total Chlorophyll *a* (Chl *a*) according to the presence and amounts of marker pigments.

Samples for flow cytometry were fixed with 25% glutaraldehyde (0.1% *v*/*v* final concentration) and immediately frozen at −80 °C until analysis. Samples were thawed for 10 min and mixed gently before measuring with an Accuri C6 flow cytometer (BD Biosciences). Autotrophic organisms were detected by their red fluorescence (fluorescence channel FL3). Using forward scatter as an indicator, three size classes were determined. Group I represented the smallest phytoplankton, approximately in the pico size range (<2 μm). Groups II and III represented phytoplankton approximately in the nano (2–20 μm) and micro size class (>20 μm), respectively. The contribution of each group to total Chl *a* fluorescence was calculated by multiplying the cell abundance within a gate and its corresponding average red fluorescence (fluorescence channel FL3).

Phytoplankton larger than 2 μm were counted using the inverted microscopy method according to Utermöhl (1958) on six different occasions during the experiment (Collet, 2013), 4 sampling days from the beginning of the experiment until maximum Chl *a* and 2 sampling days during the decline of the bloom. In order to provide a comparison to the counts obtained with the flow cytometer, diatoms were grouped according to size. Group II from the microscopy counts included diatoms <20 μm in size such as *Skeletonema costatum* and small *Chaetoceros* sp (~5 μm), while Group III included diatoms >20 μm such as *Thalassionema nitzschioides, Thalassiosira* sp, *Nitzschia longissima, Navicula transitans, Fragilaria* sp, *Guinardia* sp and *Chaetoceros* sp (>20 μm).

## Results

### CO_2_ manipulation and initial experimental conditions

**Fig. 1.**
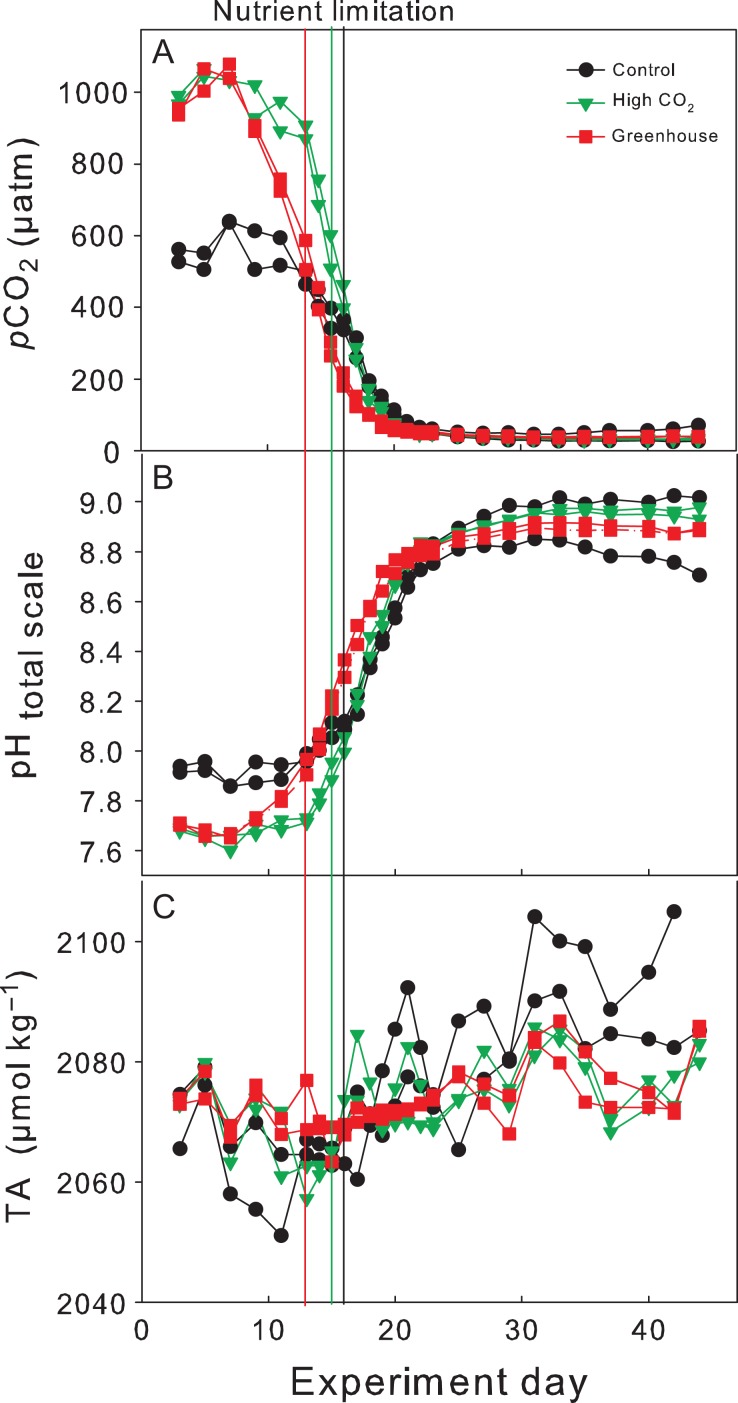
Development of (**A**) CO_2_ partial pressure (*p*CO_2_), (**B**) pH_totalscale_ and (**C**) TA during the experiment. *p*CO_2_ and pH were calculated from measured TA and DIC (see Methods for details). Vertical lines indicate time of nitrate depletion; red line corresponds to Greenhouse treatment, green line to High CO_2_ treatment and the black line to the Control.

Averaged target *p*CO_2_ levels (first 2 sampling days after CO_2_ manipulation, ±standard deviation) in the High CO_2_ and Greenhouse treatments (1020 ± 45 and 990 ± 60 μatm *p*CO_2_, respectively) were established within the first 3 days of the experiment and remained relatively constant for ~10 days. Thereafter, *p*CO_2_ levels decreased to an averaged minimum of 40 ± 10 μatm in all treatments (Fig. 1A). Starting pH_total_ values (total scale) were 7.93 ± 0.02 in the Control, 7.67 ± 0.02 in the High CO_2_ and 7.69 ± 0.02 in the Greenhouse treatment. pH_total_ increased up to maximum values of 8.98 ± 0.04 in the Control, 8.94 ± 0.02 in the High CO_2_ and 8.87 ± 0.02 in the Greenhouse treatment (Fig. 1B). TA slightly increased after nutrient depletion on Day 15 from an initial average of 2072.2 ± 3.3 μmol kg^−1^ to final concentrations of 2083.8 ± 2.4 μmol kg^−1^ (Fig. 1C). A summary of initial nutrient and *p*CO_2_ conditions as well as temperature and salinity over the experimental period is presented in Table I.

**Table I:** Summary of initial experimental conditions. Mean water temperature for each treatment throughout the experiment, initial partial pressure of CO_2_ after CO_2_ manipulation (*p*CO_2_), initial dissolved inorganic nutrient concentrations and salinity.

	Control	High CO_2_	Greenhouse
Mean water temperature (°C)	4.8 ± 0.3	5.4 ± 0.3	8.5 ± 0.4
Initial pCO_2_ (μatm)	536 ± 25	1017 ± 46	990 ± 57
Initial nutrient concentrations (μmol L^−1^)			
NO_3_^−^	11 ± 0.2		
PO_4_^−3^	0.78 ± 0.05		
Si(OH)_4_	30.0 ± 1.5		
NH_4_^+^	2.4 ± 0.2		
Salinity	20.0		

### Characterization of bloom development

**Fig. 2.**
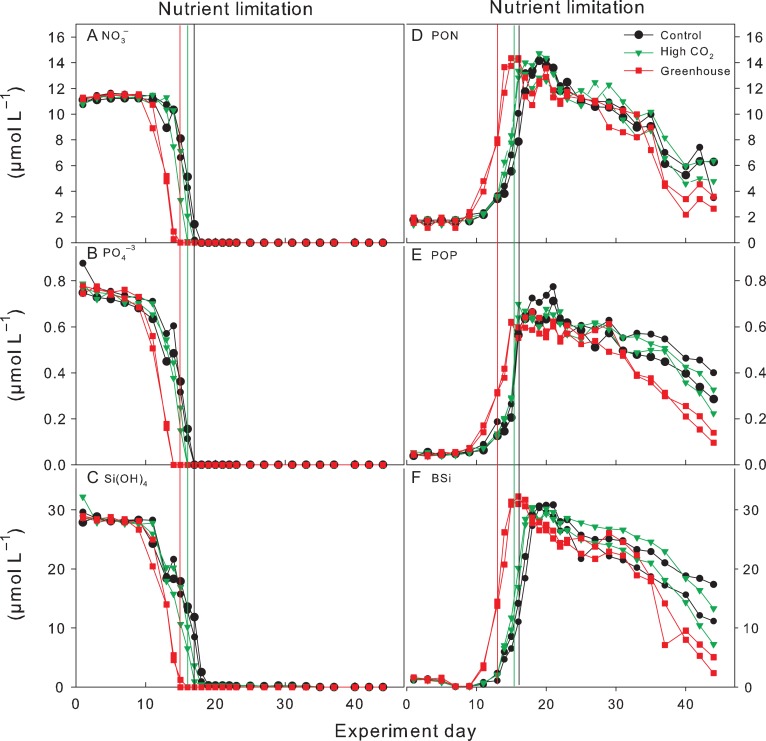
Development of major dissolved inorganic nutrients and POM concentrations during the experiment. (**A**) nitrate, (**B**) phosphate, (**C**) silicate, (**D**) particulate organic nitrogen, (**E**) particulate organic phosphorus and (**F**) biogenic silica. Vertical lines indicate time of nitrate depletion; red line corresponds to Greenhouse treatment, green line to High CO_2_ treatment and black to Control.

**Fig. 3.**
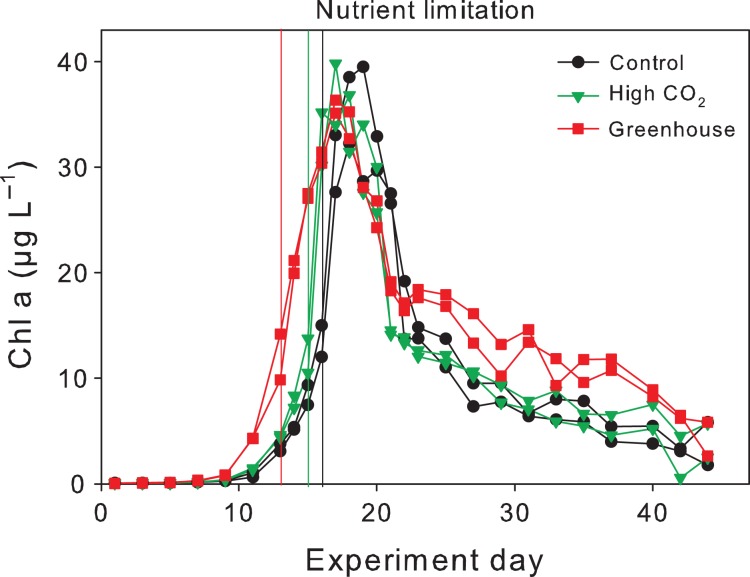
Temporal development of total Chl *a* during the experiment. Vertical lines indicate time of nitrate depletion; red line corresponds to Greenhouse treatment, green line to High CO_2_ treatment and black to Control.

Phytoplankton growth was accompanied by a rapid decrease in dissolved inorganic nutrients (Figs 2A–C and 3). Faster nutrient uptake in combination with an earlier onset of the bloom and earlier timing of maximum biomass by ~2–5 days was observed under the Greenhouse conditions, whereas overall consumption of dissolved inorganic nutrients was the same between treatments (Fig. 2A–C). In all treatments, PO_4_ was depleted 1 day earlier than any of the inorganic nitrogen species. All inorganic nutrients were exhausted by Day 15 in the Greenhouse treatment and 3–4 days later in the Control and High CO_2_. Chl *a* increased on average from ~0.09 ± 0.01 μg L^−1^ to peak average concentrations of 37 ± 2.5 μg L^−1^ in all treatments (Fig. 3). The timing of peak Chl *a* concentrations was similar among the treatments.

### Development, build-up and decline of particulate and dissolved organic matter

A concomitant increase in particulate organic matter (POM) was observed along with decreasing inorganic nutrients and increasing Chl *a.* The temporal development of PON, POP and BSi directly followed the drawdown of the corresponding inorganic nutrients (Fig. 2D–F). The onset and timing of peak concentrations was slightly earlier, ~3 days, under the Greenhouse conditions but with no differences in absolute maximum concentrations compared to the Control and High CO_2_.

PON increased from an initial average of 1.6 ± 0.22 μmol N L^−1^ to maximum average concentrations of 14.1 ± 0.65 μmol N L^−1^ in all treatments. PON concentrations at the end of the experiment were on average 5.9 ± 1.7 μmol L^−1^ in the Control, 5.8 ± 0.83 μmol L^−1^ in the High CO_2_ and 3.5 ± 0.77 μmol L^−1^ in the Greenhouse treatment.

POP increased on average from 0.05 ± 0.01 μmol P L^−1^ to maximum concentrations of 0.59 ± 0.07 μmol P L^−1^ in the Control, 0.58 ± 0.04 μmol P L^−1^ in the High CO_2_ and 0.54 ± 0.04 μmol P L^−1^ in the Greenhouse treatment. Concentrations of POP at the end of the experiment were almost twice as high in the Control (0.37 ± 0.07 μmol P L^−1^) and High CO_2_ (0.32 ± 0.07 μmol P L^−1^) compared to the Greenhouse treatment (0.15 ± 0.05 μmol P L^−1^). After PO_4_ exhaustion, DOP slightly increased to maximum concentrations of 0.16 ± 0.07 μmol P L^−1^ in all treatments and remained stable throughout the experiment with no distinct difference between treatments (data not shown).

BSi, indicative for diatom biomass, followed the development of Chl *a* from the beginning of the experiment up to the peak of the bloom. BSi increased on average from 1.0 ± 0.1 μmol L^−1^ to maximum concentrations of 28.5 ± 1.2 μmol L^−1^ in the Control, 27.9 ± 1.0 μmol L^−1^ in the High CO_2_ and 30.0 ± 0.6 μmol L^−1^ in the Greenhouse treatment. BSi concentrations remained relatively high after the peak of the bloom while the concentrations of pigments associated to diatoms decreased similarly to Chl *a* (Figs 3 and 6A). BSi comprised not only living diatoms but also silicate frustules which remained suspended in the water column for some time before sinking out. BSi concentrations at the end of the experiment were more than twice as high in the Control and High CO_2_ (14.8 ± 3.6 and 11.9 ± 3.9 μmol L^−1^, respectively) compared to the Greenhouse treatment (4.9 ± 1.9 μmol L^−1^).

### Carbon cycling

**Fig. 4.**
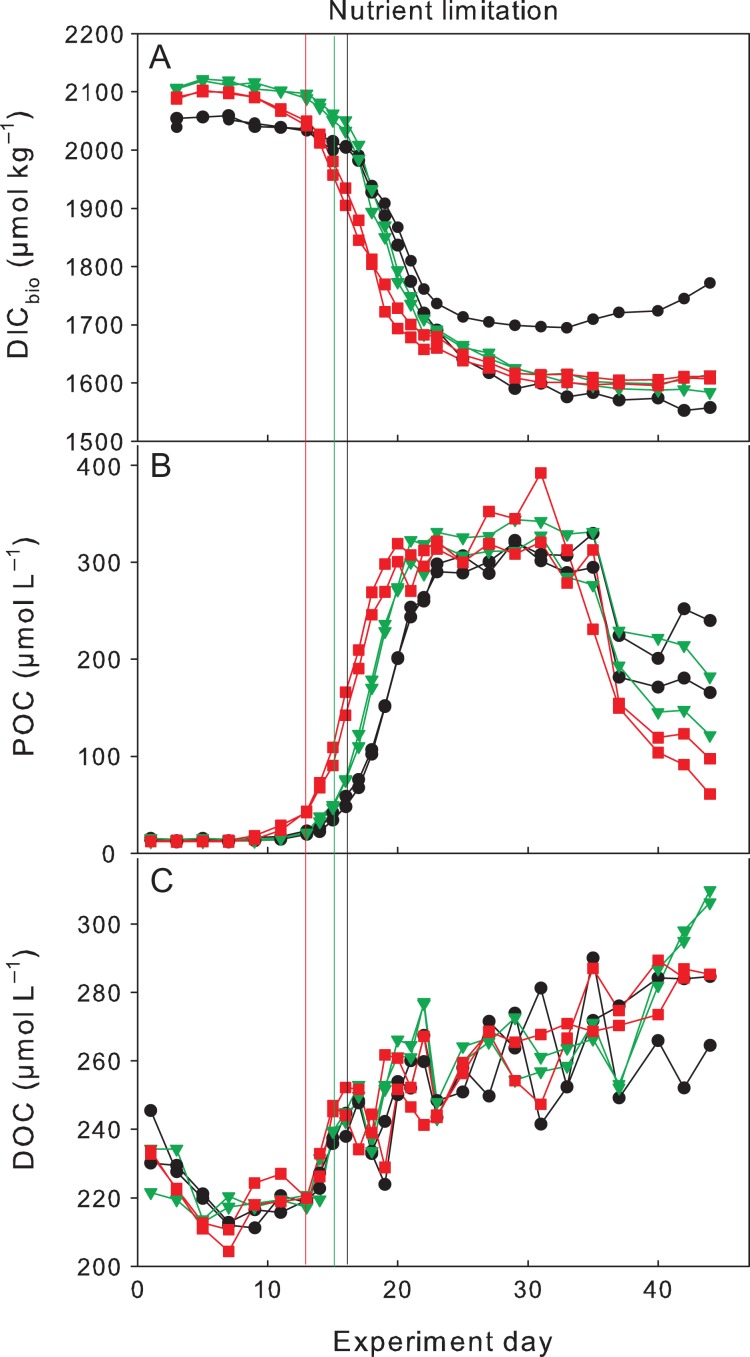
Carbon dynamics during the experiment for (**A**) DIC (corrected for gas exchange), (**B**) particulate organic carbon and (**C**) DOC. Colour coding according to Fig. 1. Vertical lines indicate time of nitrate depletion; red line corresponds to Greenhouse treatment, green line to High CO_2_ treatment and black to Control. Please note that concentrations of one of the Controls deviate from its duplicate after the bloom peak due to a defective lid (see section *Carbon cycling* in the Results section for details).

During the first part of the experiment (Days 1–20) calculated CO_2_ out-gassing did not exceed 12 μmol L^−1^ d^−1^ and during the second half of the experiment (Days 20–44) calculated CO_2_ in-gassing did not exceed 20 μmol L^−1^ d^−1^. Higher out-gassing occurred in the High CO_2_ and Greenhouse treatments, where CO_2_ gradients were larger. However, the lid in one of the Control mesocosms was not properly covering the water surface and a small opening was created near the propeller, increasing gas exchange in this mesocosm towards the end of the experiment. DIC concentrations in this Control steadily increased after Day 20 of the experiment, most likely from in-gassing, since no difference in POC or DOC concentrations was observed between this and the replicate mesocosm (Fig. 4).

Photosynthetic uptake of DIC was accompanied by an increase in POC and DOC concentrations (Fig. 4A–C). Slightly faster DIC uptake and an earlier peak in POC concentrations were observed in the Greenhouse treatment. Photosynthetic carbon fixation during the experiment decreased DIC concentrations from 2050.3 ± 9 μmol L^−1^ to 1657.6 ± 72 μmol L^−1^ in the Control and from 2113.1 ± 2 μmol L^−1^ to 1627 ± 4 μmol L^−1^ in the High CO_2_ and 2095.5 ± 1 μmol L^−1^ to 1633.4 ± 10 μmol L^−1^ in the Greenhouse treatments (Fig. 4A). A slightly higher DIC drawdown of 485.7 ± 5.8 μmol L^−1^ was calculated in the High CO_2_ compared to 389.9 ± 99 μmol L^−1^ and 462.0 ± 10 μmol L^−1^ in the Control and Greenhouse treatments, respectively. The large standard deviation in the Control stems from differences in DIC concentrations between the duplicate mesocosms due to issues with the covering lid mentioned in the previous paragraph.

POC increased from an initial average of 13.2 ± 1.1 μmol C L^−1^ to maximum concentrations of 289.7 ± 13.6 μmol C L^−1^ in the Control, 305.2 ± 15.8 μmol C L^−1^ in the High CO_2_ and 301.6 ± 26.1 μmol C L^−1^ in the Greenhouse treatment (Fig. 4B). Maximum build-up of POC occurred ~5 days after inorganic nutrient depletion and remained relatively constant for 10–15 days, then decreased rapidly towards the end. DOC steadily increased from an average of 220.6 ± 7.3 μmol C L^−1^ to maximum concentrations of 271.3 ± 16 μmol C L^−1^ in the Control, and 285.4 ± 1.3 μmol C L^−1^ in the Greenhouse and slightly higher in the High CO_2_ treatments with 302.3 ± 7 μmol C L^−1^ at the end of the experiment (Fig. 4C).

### Elemental stoichiometry of POM

**Fig. 5.**
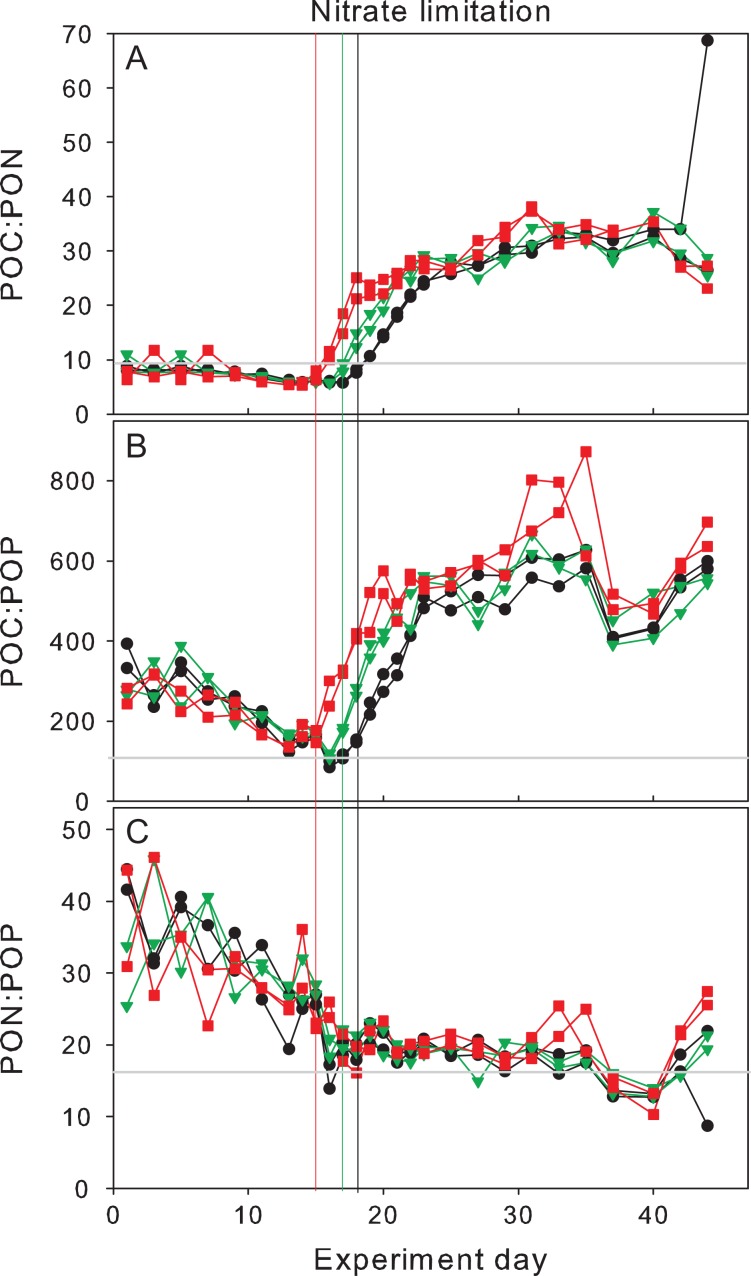
Elemental stoichiometry of POM during the experiment. Colour coding according to Fig. 1. Vertical lines indicate time of nitrate depletion; red line corresponds to Greenhouse treatment, green line to High CO_2_ treatment and black to Control. Grey horizontal lines represent Redfield proportions of (**A**) 6.6, (**B**) 106:1 and (**C**) 16:1.

Inorganic nutrients were assimilated into the respective POM pools according to Redfield proportions up to Day 15 of the experiment. Thereafter, due to nutrient limitation, POC:PON and POC:POP increased strongly, while PON:POP remained relatively stable (Fig. 5). Both POC:POP and PON:POP started at values almost twice as high as the respective Redfield ratio (106:1 and 16:1), suggesting a low contribution of living phytoplankton biomass to the POM pool at the beginning of the experiment (Fig. 5B and C). Carbon fixation continued after nutrient depletion, leading to maximum POC:PON of up to 30–40 and POC:POP of up to 600–800. Except for the faster bloom onset in the Greenhouse treatment, no differences in maximum ratios were observed between the treatments (Fig. 5A–C).

### Phytoplankton composition derived from HPLC analysis

**Fig. 6.**
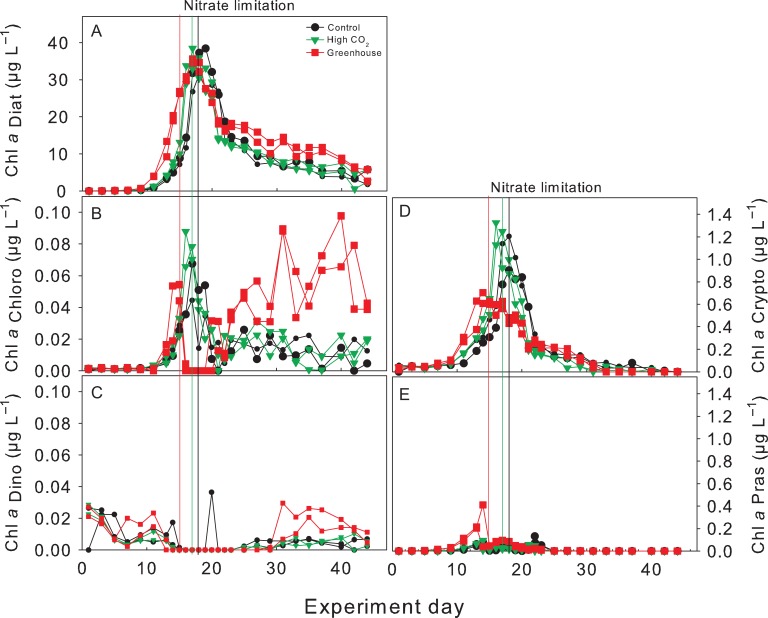
Chl *a* equivalent concentrations of: (**A**) Diatoms, (**B**) Chlorophytes, (**C**) Dinophytes, (**D**) Cryptophytes and (**E**) Prasinophytes analysed by CHEMTAX. Vertical lines indicate time of nitrate depletion; red line corresponds to Greenhouse treatment, green line to High CO_2_ treatment and black to Control.

Chl *a* was almost exclusively produced by diatoms with minor contributions from cryptophytes, prasinophytes, dinophytes and chlorophytes (Fig. 6A–E). Diatoms contributed up to ~35 μg L^−1^ to the total average of 37 ± 3 μg L^−1^ Chl *a* (Fig. 6A). Cryptophytes were the second group after diatoms contributing up to ~1.2 μg L^−1^ to Chl *a* while the minor contributions from the other phytoplankton groups did not exceed ~0.5 μg L^−1^ (Fig. 6B–E). Contributions of prasinophytes were higher in the Greenhouse treatment compared to the Control and High CO_2_ treatments while the opposite was observed for cryptophytes showing twice the concentrations in the Control and High CO_2_ compared to the Greenhouse treatment (Fig. 6D and E). Chl *a* contributions of chlorophytes and dinophytes showed a similar response, increasing after the peak of the bloom with higher concentrations in the Greenhouse treatment (Fig. 6B and C), though at relatively low-absolute concentrations.

### Phytoplankton composition based on flow cytometry and microscopy

**Fig. 7.**
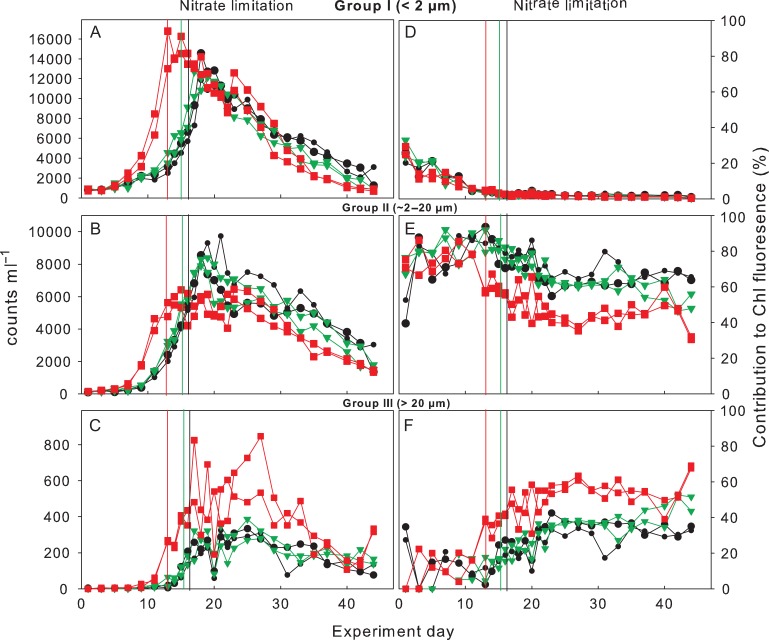
Cell counts according to group sizes from flow cytometry (left panel): (**A**) Group I (<2 μm), (**B**) Group II (2–20 μm) and (**C**) Group III (>20 μm) and percent contribution to total Chlorophyll fluorescence (right panel): (**D**) Group I, (**E**) Group II and (**F**) Group III. Colour coding according to Fig. 1. Vertical lines indicate time of nitrate depletion; red line corresponds to Greenhouse treatment, green line to High CO_2_ treatment and black to Control.

Phytoplankton abundances as measured by flow cytometry increased to peak concentrations similarly to the development of Chl *a* (Fig. 7A–C). Abundances in Group I (<2 μm) decreased after reaching peak concentrations between Days 15 and 18 while abundances in Group II (~2–20 μm) and Group III (>20 μm) remained relatively stable for ~10–15 days. The contribution of Group I to total Chl fluorescence (i.e. cell abundances multiplied by average red fluorescence) at the beginning of the experiment was up to 30% but steadily decreased towards the peak of the bloom, accounting for <2% thereafter (Fig. 7D). Group II showed peak abundances 1 day after nutrient depletion. Abundances of Group II were ~25% lower in the Greenhouse treatment compared to those in the Control and High CO_2_ treatments (Fig. 7B). The contribution of Group II to Chl fluorescence during the bloom peak was >60% in the Control and High CO_2_ and ~45–50% in the Greenhouse treatment (Fig. 7E). Conversely, Group III showed higher abundances in the Greenhouse treatment during peak Chl *a* (and thereafter) compared to the Control and High CO_2_ treatments (Fig. 7C). The contribution to Chl fluorescence during peak concentrations by Group III was ~50% in the Greenhouse treatment and <40% in the Control and High CO_2_ (Fig. 7F). Since more than 95% of the Chl could be attributed to diatoms from the peak of the bloom towards the end of the experiment, Groups II and III as identified by flow cytometry are most likely primarily composed of diatoms.

Although absolute values were not the same between microscopic and flow cytometry counts, a tendency towards larger diatom species in the Greenhouse treatment was observed with both methods. The shift towards larger diatoms in the Greenhouse treatments was most likely driven by the increased abundances of *Thalassiosira* sp, *Thalassionema nitzschioides* and *Nitzschia longissima* (Table II).

**Table II:** Summary of microscopic counts of diatom species for Groups II and III. Mean abundances represent the average of the duplicates on the specific experiment day ±standard deviation.

Experiment day	1	11	16	17	19	31	44
Group II (mean abundance ± SD)							
*Chaetoceros* sp ~5 μm							
Control	–	6 ± 5	39 ± 18	37 ± 23	37 ± 18	8 ± 5	5 ± 1
High CO_2_	–	4 ± 3	28 ± 3	16 ± 1	28 ± 11	11 ± 9	–
Greenhouse	–	5 ± 4	25 ± 18	15 ± 5	14 ± 1	2 ± 1	–
*Skeletonema costatum*							
Control	2 ± 1	67 ± 30	1032 ± 1013	5686 ± 1727	2245 ± 1282	3989 ± 1474	682 ± 453
High CO_2_	1 ± 0.5	106 ± 22	1487 ± 60	2430 ± 309	3019 ± 2659	1929 ± 357	25 ± 1
Greenhouse	1 ± 0.5	349 ± 109	2393 ± 670	3456 ± 2410	2833 ± 511	516 ± 124	–
Group III (mean abundance ± SD)							
*Chaetoceros* sp > 20 μm							
Control	–	–	62 ± 9	94 ± 27	73 ± 23	106 ± 29	66 ± 14
High CO_2_	–	10 ± 7	57 ± 15	72 ± 11	107 ± 16	72 ± 51	34 ± 19
Greenhouse	–	19 ± 1	115 ± 15	95 ± 23	74 ± 21	35 ± 11	–
*Thalassiosira* sp							
Control	–	28 ± 3	305 ± 156	730 ± 50	657 ± 153	695 ± 56	383 ± 89
High CO_2_	–	42 ± 4	440 ± 41	960 ± 23	784 ± 149	655 ± 357	398 ± 69
Greenhouse	1 ± 1	119 ± 38	1054 ± 80	1022 ± 257	1061 ± 283	1116 ± 357	174 ± 101
*Thalassionema nitzschioides*							
Control	–	3 ± 1	12 ± 9	12 ± 1	23 ± 7	25 ± 5	–
High CO_2_	–	–	18 ± 8	18 ± 2	13 ± 1	5 ± 2	–
Greenhouse		7 ± 4	45 ± 20	37 ± 5	41 ± 22	33 ± 17	–
*Nitzschia longissima*							
Control	–	6 ± 1	15 ± 4	28 ± 5	18 ± 1	24 ± 4	34 ± 18
High CO_2_	–	4 ± 1	23 ± 2	31 ± 3	23 ± 6	37 ± 10	27 ± 9
Greenhouse	–	44 ± 4	271 ± 98	289 ± 23	393	335 ± 27	148 ± 141
*Navicula transitans*							
Control	–	3 ± 1	15 ± 3	26 ± 17	49 ± 3	73 ± 11	22 ± 2
High CO_2_	–	7 ± 4	13 ± 11	39 ± 20	55 ± 28	85 ± 21	6 ± 3
Greenhouse	–	11 ± 2	26 ± 8	32 ± 3	38 ± 16	24 ± 6	–
*Fragilaria* sp							
Control	–	4 ± 3	38 ± 27	71 ± 20	57 ± 5	57 ± 6	18 ± 9
High CO_2_	–	–	15 ± 11	43 ± 36	47 ± 17	17 ± 15	1 ± 1
Greenhouse	–	13 ± 7	42 ± 23	33 ± 3	38 ± 28	23 ± 6	–
*Guinardia* sp							
Control	–	2 ± 1	94 ± 18	179 ± 63	203 ± 116	277 ± 71	318 ± 133
High CO_2_	–	6 ± 4	145 ± 69	421 ± 186	69 ± 49	868 ± 90	324 ± 74
Greenhouse	–	12 ± 1	140 ± 35	178 ± 6	115 ± 81	105 ± 51	4 ± 1

Microzooplankton abundances in our experiments (comprising flagellates, dinoflagellates and ciliates) increased at the beginning of the experiment up to the peak of the bloom and decreased rapidly thereafter, similarly to the results reported by Lewandowska and Sommer (2010), showing no difference between treatments.

## Discussion

Ocean acidification and warming have the potential to significantly affect marine biogeochemical element cycling by modifying the balance between primary production and heterotrophic processes and/or changing plankton community composition (Riebesell and Tortell, 2011). Our study examined the combined effects of these two drivers on the species composition and biogeochemical dynamics during a spring phytoplankton bloom with a natural plankton community from a temperate coastal ecosystem in the Baltic Sea. The majority of experiments in this region focused on the individual effects of either warming (Wohlers-Zöllner *et al.*, 2012; Sommer *et al.*, 2012b) or CO_2_ (Schulz and Riebesell, 2013; Engel *et al.*, 2014). So far only the study by Paul *et al.* (2015) examined the combined influence of both drivers on a natural autumn Baltic Sea plankton assemblage.

### Phytoplankton community composition and shifts in size

A phytoplankton bloom, dominated by diatoms (98% of total Chl *a*), developed in our experiments under all treatment conditions (Fig. 6). The chain-forming diatom *S. costatum* typically dominates spring blooms in the Kiel Bight (Wasmund *et al.*, 2008) and was also the most abundant species under all treatment conditions during our experiments (60–70% of diatom abundance). There is significant evidence from the field showing that over the past 50 years, diatom abundance positively correlated with warming and increasing wind conditions in the North Sea (Hinder *et al.*, 2012). Phytoplankton biomass almost doubled over the past century with diatoms contributing to up to 80% of the carbon biomass during spring blooms in the Kiel Bight, the same region of the present study (Wasmund *et al.*, 2008). Both of these studies also reported increasing abundances of *Skeletonema* spp, the dominating group on our experiments. However, in our experiments we observed a shift towards larger diatom species (mainly *Thalassiosira* sp and *Nitzschia longissima*, Table II) in the Greenhouse treatment. Due to experimental constraints we did not include a “warming only” treatment in our design. Thus, we could not disentangle the influence of warming and acidification individually on this phytoplankton community. Nonetheless, in the following section we will discuss whether the individual or the combined effect of both stressors could have driven the shift in phytoplankton size in the Greenhouse treatment of our experiments.

The shift towards larger diatom species was somewhat contrary to previous studies which reported that warming alone generally decreased abundances of large diatom species, reduced cell size and benefited smaller phytoplankton (Sommer and Lengfellner, 2008a; Daufresne *et al.*, 2009; Lewandowska and Sommer, 2010; Morán *et al.* 2010). However, the increase of small-sized phytoplankton in the latter experiments was mostly associated with higher grazing rates of zooplankton on larger phytoplankton species allowing smaller ones to proliferate (Keller *et al.*, 1999; O’Connor *et al.*, 2009; Sommer and Lewandowska, 2010). Furthermore, the addition of adult copepods in those experiments might have generated a selective grazing pressure on larger phytoplankton since both copepods and ciliates in this region have shown to preferentially feed on larger phytoplankton during their adult lifetime (Sommer *et al.*, 2002; Sommer and Lengfellner, 2008b; Aberle *et al.*, 2007).

While physiological studies with single species give valuable information on tolerance ranges and help us understand underlying mechanisms for the response of species to different stressors (Torstensson *et al.*, 2013; Li *et al.*, 2018), these results cannot be extrapolated to how a species would respond when being part of a more complex (mixed) plankton community. During CO_2_ enrichment experiments with natural phytoplankton communities, both positive and negative effects of increasing CO_2_ were observed on a number of functional groups and species. For instance, small picoeucaryotes profited from higher CO_2_ levels (Yoshimura *et al.*, 2013; Crawfurd *et al.*, 2017; Schulz *et al.*, 2013, 2017). The specific mode of carbon concentrating mechanism (CCM) employed for inorganic carbon acquisition was speculated to be among the reasons for this response, relying more on passive diffusion in very small cells. However, not only relatively small but also relatively large phytoplankton cells have been found to directly profit from higher CO_2_ concentrations. Incubation experiments with natural Southern Ocean phytoplankton communities showed that large chain-forming *Chaetoceros* sp profited at high-CO_2_ conditions at the expense of smaller diatoms such as *Pseudo-nitzschia subcurvata* and *Cylindrotheca closterium* (Tortell *et al.*, 2008; Feng *et al.*, 2010). Similarly, Wu *et al.* (2011) showed that although increasing CO_2_ consistently enhanced growth rates of five different diatom species ranging in size, the largest enhancement (i.e. 33% compared to 5%) was observed for the largest species. This was hypothesized to be related to an alleviation of carbon limitation in larger cells which have a thicker diffusive boundary layer compared to smaller cells.

Incubation experiments with natural Bering Sea phytoplankton communities revealed either a negative or no effect of elevated CO_2_, increased temperature or the combination of both on diatom cell numbers in comparison to a control (Hare *et al.*, 2007). A positive effect of elevated CO_2_ alone, but not in combination with increased temperature, was observed in a North Atlantic community (Feng *et al.*, 2009). In the latter experiment, however, it is important to note, that nutrient levels applied with silicate to nitrate ratios of about 0.15 are rather disadvantageous for diatom growth, also indicated by lower diatom biomass towards the end of the experiment in most incubations compared to the beginning. Also, a shift towards larger species within the same functional group (i.e. diatoms), as observed in our experiment, is not easily detected by looking at overall abundances. For example, Feng *et al.* (2009) reported an increase in overall diatom abundance only in the High CO_2_ treatment but the shift towards larger species (i.e. *Pseudo-nitzschia* to *Cylindrotheca* ratio) was more pronounced in the combined treatment (“Greenhouse”), although the absolute diatom abundances were three times lower than in the High CO_2_. Additionally, incubation experiments with a natural plankton community from the subtropical North Atlantic showed that if the community was initially dominated by diatoms, elevated CO_2_ tended to favour the larger diatom species (Eggers *et al.* 2014).

### Temporal development of the bloom and POM dynamics

The temporal development of POM during the nutrient replete phase of the experiment (Days 1–15) followed a typical natural diatom-dominated spring bloom exhibiting rapid drawdown of inorganic nutrients and accompanied by exponential autotrophic growth (as represented by Chl *a*) and biomass build-up of POC, PON, POP, and BSi. Once inorganic nutrients were exhausted between Days 15 and 18 (depending on treatment), peak concentrations in the respective particulate pools were reached (Fig. 2). During the first part of the experiment (i.e. exponential growth phase), higher temperature in the Greenhouse treatment accelerated nutrient uptake and caused maximum POM concentrations being reached ~2–5 days earlier compared to the Control and the High CO_2_ treatments. Such a response (i.e. earlier onset of the bloom and faster biomass build-up) was consistently observed in mesocosm experiments investigating warming (Sommer *et al.*, 2012a) and is most likely due to enhanced metabolic activities at higher temperature. Even so, a week or less (2–3 days) acceleration of such phytoplankton bloom events (i.e. less time from bloom initiation to bloom peak) in nature would be negligible and does not seem to be of present concern based on long-term monitoring records on spring bloom regions (Wiltshire *et al.*, 2008).

Peak concentrations of PON, POP and BSi were observed 1 day after the respective inorganic nutrients were depleted with no differences in maximum POM concentrations between the treatments (Fig. 2 D–F). Mesocosm experiments investigating the impact of warming on plankton communities from the Kiel Bight consistently yielded a decrease in phytoplankton biomass with increasing temperature during spring blooms (Sommer and Lengfellner 2008; Sommer *et al.*, 2012b) and autumn blooms (Paul *et al.*, 2015) with only one study showing the opposite response during a summer bloom (Taucher *et al.*, 2012). Taucher *et al.* (2012) suggested that the contrasting results were most likely due to differences in the phytoplankton assemblage. While most of the spring experiments were dominated by the typical “spring bloom” species *S. costatum*, the summer experiment by Taucher *et al.* (2012) was dominated by the diatom *Dactyliosolen fragilissimus.*

DIC uptake and POC build-up continued for another 5 days after nutrient depletion and thereafter POC concentrations remained constant for ~15 days while DIC concentrations remained low (Fig. 4B–C). Chl a, however, declined sharply directly afterwards (Fig. 3). These results are contrasting to previous warming experiments where POM concentrations were more closely coupled to Chl *a* and decreased immediately after peak concentrations (Wohlers *et al.*, 2009; Biermann *et al.*, 2014). In the latter experiments, however, mesozooplankton (i.e. copepods) was added after initial filling of the mesocosms to investigate the influence of warming on grazing activities. Therefore, phytoplankton growth was top-down controlled in these experiments and biomass build-up restricted (Sommer and Lewandowska, 2010; Lewandowska *et al.*, 2014). The lower grazing pressure in our experiment favoured fast autotrophic biomass build-up in all treatments followed by a phase of relatively stable POM concentrations for 10–15 days after the bloom peak (Fig. 2D, E and F). Relatively stable POM concentrations after the bloom peak were also observed in similar experiments with nutrient induced diatom blooms under low-grazing pressures (Engel *et al.*, 2002; Wohlers-Zöllner *et al.*, 2011). As in our experiment, post-bloom conditions most likely lead to the retention of diatom aggregates in the water column (Smayda and Boleyn, 1966; Sarthou *et al.*, 2005).

Towards the end of the experiment and after the phase of stable POM concentrations, the loss of POM in the Greenhouse treatment was about twice as fast as in the Control and High CO_2_ treatments. As this loss of POM did not correspond to an increase in any of the dissolved matter (both organic and inorganic) pools, faster degradation at elevated temperature was unlikely the cause. Thus, two possible mechanisms remain that could explain these results, either the initiation of aggregation processes and therefore the earlier sinking/loss of POM from the water column (Piontek *et al.*, 2009), was driven by higher temperatures or by the accompanied phytoplankton community shift towards larger diatoms.

Diatoms are known to be important organic matter exporters as they often contribute a high fraction of the biomass produced during spring blooms (Buesseler, 1998; Sarthou *et al.*, 2005 and references therein). Boyd and Newton (1995) followed the development of the spring bloom in 2 consecutive years in the North East Atlantic and showed that although primary production was comparable in both years the export of organic carbon was more efficient, from the surface to the deep ocean, when the phytoplankton assemblage was dominated by large diatom species. Thus, the higher abundance of larger diatoms in our experiments could have been a crucial player in determining faster loss processes in the Greenhouse treatment. However, while cell-specific sinking rates between small and large cells might have been relevant in our 1 m deep tanks, extrapolation of our results to sinking rates in nature are to be made cautiously.

## Conclusion

It was recently suggested that the interactive effects of environmental drivers, rather than the sum of the individual effects, best explained plankton responses in aquatic ecosystems (Christensen *et al.*, 2006). In our experiment with a natural phytoplankton assemblage from a coastal eutrophic ecosystem, we observed that in the combined ocean acidification and warming treatment a shift in size towards larger diatoms was favoured. This shift was not observed in the High CO_2_ treatment and while we did not have a warming treatment alone, based on evidence from similar experiments investigating warming and CO_2_ separately, we hypothesize that the combination of both factors was important for the shift in size. The potential for warming and CO_2_ induced changes in species composition and size distribution during bloom events in the future ocean is likely to influence biogeochemical element cycling with consequences for CO_2_ partitioning between ocean and atmosphere.
